# A novel non-invasive diagnostic approach based on urine and blood: a cross-sectional study combining lipoarabinomannan antigen and interferon-gamma release assay for active tuberculosis

**DOI:** 10.3389/fimmu.2025.1726930

**Published:** 2026-01-09

**Authors:** Long Cai, Mingzhi Zhu, Tingting Fang, Qianqian Peng, Lingshan Dai, YanQin Shao, Libin Liu

**Affiliations:** Centre of Laboratory Medicine, Hangzhou Red Cross Hospital, Hangzhou, Zhejiang, China

**Keywords:** interferon-gamma release assays, lipoarabinomannan, parallel testing, serial testing, tuberculosis

## Abstract

**Purpose:**

To evaluate the diagnostic value of a combined strategy using the urine lipoarabinomannan (LAM) antigen assay and interferon-gamma release assay (IGRA) for active tuberculosis (TB), and to explore its potential as a non-invasive, sputum-independent diagnostic method for clinical application.

**Patients and Methods:**

A cross-sectional study design was adopted. Initially, 313 valid cases were selected for LAM performance evaluation, subsequently, 142 cases with both valid LAM and IGRA results were further selected from this group for combined test performance analysis. Using comprehensive clinical diagnosis as the reference standard, the diagnostic performance of LAM, IGRA, and two combination strategies (parallel: positive if either LAM or IGRA is positive; serial: positive only if both are positive) was evaluated, and their distribution characteristics in differentiating between tuberculosis and nontuberculous mycobacterial (NTM) infection were preliminarily explored.

**Results:**

The urine LAM assay demonstrated a sensitivity of 72% and a specificity of 79% for active TB. Its sensitivity was higher than that of smear microscopy (33%) and culture (56%), and slightly superior to Xpert MTB/RIF (69%) and TB-DNA (65%), although its specificity was lower. IGRA showed high sensitivity (92%) but moderate specificity (59%). The combination of LAM and IGRA significantly improved diagnostic performance: parallel testing achieved a sensitivity of 98%, making it suitable for TB screening and rule-out purposes, while serial testing increased specificity to 91%, supporting the confirmatory diagnosis of active TB. Among patients with NTM infection, the LAM^+^/IGRA^−^ pattern accounted for 50% of cases, suggesting its potential utility in discriminating between TB and NTM infection.

**Conclusion:**

The combination of urine LAM antigen testing and blood-based IGRA optimizes TB diagnosis by simultaneously targeting two distinct dimensions: pathogen-derived antigens and host immune response. The parallel testing improves sensitivity for screening, while the serial testing enhances specificity for confirmatory diagnosis. This strategy offers a non-invasive approach with ease of sample collection, demonstrating particular value in diagnosing challenging cases such as sputum smear-negative pulmonary TB, extrapulmonary TB, and TB in immunocompromised patients.

## Introduction

*Mycobacterium tuberculosis* (*M. tuberculosis*), the causative agent of a chronic infectious disease known as tuberculosis (TB), remains a major global public health challenge. According to the 2024 World Health Organization (WHO) report, approximately 10.8 million individuals developed TB globally in 2023, reestablishing TB as the leading infectious cause of mortality worldwide (1). The persistent spread of the epidemic is largely attributable to the limitations of current TB diagnostic technologies. The sensitivity of conventional pathogen-based detection methods remains notably compromised in specific populations, including immunocompromised individuals, patients with paucibacillary or sputum-scarce disease, and those with extrapulmonary tuberculosis (EPTB) (2). Currently, conventional methods such as smear microscopy and mycobacterial culture remain widely used but are limited by low sensitivity and lengthy turnaround times (3). While the introduction of molecular diagnostic techniques has significantly improved TB diagnostic performance in recent years (4), these methods still heavily rely on pathogen detection in clinical samples. Consequently, their diagnostic utility remains suboptimal for patients who have difficulty producing adequate sputum specimens. In response, the WHO has explicitly urged the accelerated development of rapid, non-sputum-based diagnostic tests and has identified this technology as a priority target product profile for global TB diagnosis (5).

Against this backdrop, diagnostic strategies based on host immune responses have gained increasing attention. The interferon-gamma release assay (IGRA) evaluates the infection status of *M. tuberculosis* by detecting interferon-gamma (IFN-γ) levels produced by T cells in response to stimulation with *M. tuberculosis*-specific antigens (such as ESAT-6 and CFP-10). This method demonstrates high sensitivity and has been widely adopted for population-level screening of TB infection (6, 7). However, IGRA has notable limitations: it cannot reliably differentiate between active TB and latent infection, and its sensitivity is significantly reduced in immunocompromised individuals (8). These shortcomings substantially limit its clinical utility in high-risk populations.

Simultaneously, diagnostic approaches based on direct detection of pathogen antigens have demonstrated significant potential. Lipoarabinomannan (LAM), a key component of the *M. tuberculosis* cell wall, can be released into the bloodstream during bacterial metabolism or lysis and is subsequently filtered by the kidneys into urine, thereby establishing it as an easily accessible, non-invasive diagnostic biomarker (9–11). Notably, the detection performance of LAM is highly dependent on the host’s immune status. The Alere Determine™ TB LAM Ag assay, currently recommended by the WHO, is primarily indicated for use in HIV-positive TB patients with severe immunosuppression (12). Although the application of newer technologies such as chemiluminescence has significantly improved the sensitivity of LAM detection, its diagnostic performance still varies considerably across populations with different immune backgrounds (13).

Therefore, urine-based LAM assay (reflecting pathogen antigen presence) and blood-based IGRA (detecting specific cellular immune responses) provide complementary insights into TB infection from distinct immunological dimensions: LAM demonstrates higher detection sensitivity in immunocompromised patients, while IGRA, currently used for the detection of *M. tuberculosis* infection, maintains more consistent performance in immunocompetent individuals. The two methods are therefore theoretically highly complementary, particularly in challenging clinical scenarios such as active TB with immunosuppression, smear-negative pulmonary tuberculosis(PTB), and EPTB. In these contexts, the combined use of LAM and IGRA testing can help overcome the limitations of single-method approaches and significantly enhance overall diagnostic accuracy.

This cross-sectional study aims to systematically evaluate the diagnostic value of combining urine LAM testing with IGRA in patients with active TB. We will compare the diagnostic performance of this combined strategy against conventional microbiological methods and preliminarily explore its potential in differentiating between TB and non-tuberculous mycobacterial (NTM) infections. The findings are expected to provide a theoretical foundation and evidence-based support for developing a new, efficient, non-invasive TB diagnostic strategy grounded in immune mechanisms.

## Materials and methods

### Study design and populations

This cross-sectional study consecutively enrolled 344 patients with suspected TB from Hangzhou Red Cross Hospital between April 2023 and June 2024. All enrolled patients underwent urine LAM test using chemiluminescence immunoassay, and systematic collection of TB-related laboratory results was performed, including acid-fast staining, mycobacterial culture, Xpert MTB/RIF (Xpert), and IGRA test. The definitive diagnosis of active TB was established based on the comprehensive criteria outlined in the “WS 288-2017 Diagnostic Criteria for Pulmonary Tuberculosis” (14). Patients confirmed with TB were stratified into three categories: PTB, EPTB, and those with concurrent PTB and EPTB (PTB/EPTB). For PTB, a definitive diagnosis was established primarily through etiological confirmation, defined as: a positive *Mycobacterium tuberculosis* nucleic acid amplification test; or one positive sputum smear plus a culture confirmed as the *M. tuberculosis* complex. Cases lacking positive bacteriology but meeting specific clinical and radiological criteria, supported by a positive IGRA result and after the exclusion of alternative diagnoses, were classified as clinically diagnosed cases. For EPTB, diagnosis was confirmed by the histopathological identification of epithelioid cell granulomas with caseous necrosis in biopsy specimens, with or without a positive etiological test on the tissue. The control group comprised patients with NTM infections and those with other non-tuberculous disease (Non-TB). All control subjects were ruled out through microbiological testing, received clear alternative diagnoses, and were confirmed to have improved without anti-TB treatment after a follow-up period of at least 2 months, thereby minimizing the risk of false negatives.

Regarding the Implementation of Blinding: 1) Blinding in the Index Tests: Laboratory personnel processing the urine LAM and blood IGRA tests had access only to anonymized codes and were unaware of all patient clinical information, group assignments, and other test results. All testing procedures and result interpretations strictly followed the reagent instructions. Key indicators (such as LAM concentration and IFN-γ levels) were automatically calculated by instruments and determined based on predefined criteria, thereby eliminating human intervention and subjective bias in both operational and interpretative steps. 2) Blinding in the Reference Standard: The final clinical diagnoses for all patients were independently established by clinical experts not involved in this study, strictly adhering to the WS 288–2017 standards. This approach severed the influence of research data on diagnostic judgment at the source, ensuring the objectivity and reliability of the reference standard.The study protocol was approved by the Ethics Committee of Hangzhou Red Cross Hospital (Approval No.: 2023YS057), and all study procedures strictly adhered to the ethical principles of the World Medical Association’s Declaration of Helsinki.

### Patient selection

Suspected TB patients included individuals presenting for initial consultation or referred to our hospital with clinical features suggestive of active TB but without confirmed diagnosis, or those requiring further differential diagnosis despite previous treatment (15). All participants enrolled in this study were confirmed to be HIV-negative through serological testing. Inclusion criteria comprised: (1) complete demographic information, laboratory test data, and clinical documentation; (2) availability of valid urine LAM results. Exclusion criteria were: (1) cases with indeterminate final diagnosis; (2) duplicate enrollments or repeated testing from the same patient; (3) patients with *M. tuberculosis*-NTM co-infection; (4) those who had received anti-TB treatment for more than 2 weeks. Combined LAM/IGRA Testing: Parallel testing was defined as a positive result in either the LAM assay or the IGRA being considered a positive overall result; Serial testing was defined as a positive result in both the LAM assay and the IGRA being required for a positive overall result.

### Mycobacterial culture

Mycobacterial culture was performed using the automated Bactec MGIT 960 system (Becton Dickinson, USA) in accordance with the manufacturer’s instructions.

### IGRA

A total of 3-5 mL of heparin-anticoagulated venous blood were collected, and the IGRA assay was performed using a commercial kit (Raide Biotechnology Co., Ltd., China, http://www.leidebio.com/) according to the manufacturer’s instructions. Samples that were hemolyzed, lipemic, or hyperbilirubinemic were excluded. Whole blood was aliquoted into stimulation tubes coated with TB-specific antigens, a positive control (phytohemagglutinin), and a negative control. Following incubation at 37°C for 20 hours, the samples were centrifuged to harvest plasma. The concentration of IFN-γ in the plasma was measured using a microplate reader at a wavelength of 450/630 nm. According to the manufacturer’s criteria, a result was considered positive if the IFN-γ level in the antigen-stimulated tube minus that of the negative control (T-N) was ≥20 pg/mL and also ≥25% of the negative control value. All procedures were strictly performed in accordance with the manufacturer’s protocols and standard laboratory guidelines.

### LAM assay

This assay employs a double-antibody sandwich chemiluminescence method. The LAM capture antibody-coated magnetic beads bind to the LAM antigen in urine samples, forming a complex with the acridinium ester-labeled detection antibody. The resulting chemiluminescence signal intensity is directly proportional to the LAM concentration. For this assay, the following sample collection and handling procedures were followed: Clean-catch midstream urine samples with a minimum volume of 5 mL were collected, avoiding specimens with high protein or lipid content. The samples were stored at -80°C, avoiding repeated freeze-thaw cycles. For testing, 4 mL of each sample was transferred to a 5 mL centrifuge tube, followed by the addition of 85 μL of uniformly mixed antibody-coated magnetic beads. The mixture was incubated at room temperature for 2 hours with continuous rotation at 30 rpm (Roller Mixer, RM100, https://www.ybzhan.cn/st123573/). After incubation, magnetic separation was performed using a magnetic rack to immobilize the beads, and the supernatant was carefully discarded. The beads were then resuspended in 2 mL of sample dilution buffer and vortexed thoroughly. This washing procedure was repeated once. Finally, 200 μL of dilution buffer was added, and the mixture was vortexed to resuspend the beads, while avoiding bead adherence to the tube wall or bubble formation. The suspension was then transferred to a 2 mL detection tube and thoroughly mixed. Measurement was performed within 5 minutes using the SMART500S fully automated chemiluminescence immunoanalyzer (Chongqing KESMAI Biotechnology Co., Ltd. China) and the matched LAM antigen detection kit (Guangzhou Reade Biotechnology Co., Ltd. China). The SMART500S analyzer automatically adds acridinium ester-labeled detection antibodies to form immunocomplexes, subsequently initiating chemiluminescence detection. All procedures were strictly conducted in accordance with the manufacturer’s instructions (http://www.leidebio.com/invitrodiagnostics/info.aspx?itemid=509).

### Real-time PCR for M. tuberculosis (TB-DNA)

The detection of *Mycobacterium* nucleic acid was performed using a PCR-fluorescent probe method kit from CapitalBio Technology Inc (https://www.capitalbiotech.com/), based on a dual real-time PCR technology. This technique employs TaqMan probes labeled with FAM and HEX fluorescent dyes, and the assay was run on an SLAN-96P real-time PCR system. Clinical samples were subjected to liquefaction, washing, and DNA extraction prior to PCR amplification.

Result interpretation was based on the Ct values from the FAM and HEX channels: a sample was considered positive if an S-shaped amplification curve was observed in either channel with a Ct value < 40. Specifically, positivity in the FAM channel indicated *Mycobacterium tuberculosis* complex, while positivity in the HEX channel indicated NTM. If no amplification was observed in either channel, the result was interpreted as negative for *Mycobacterium* nucleic acid.

### Statistical analysis

For continuous data with skewed distributions, median (M) and interquartile range (Q1, Q3) were used to describe central tendency and dispersion. Group comparisons were performed using the Mann–Whitney U test. Categorical data were presented as number (n) and percentage (%), and group differences were assessed using the chi-square test or Fisher’s exact test, as appropriate.

Diagnostic performance was evaluated using composite clinical diagnosis as the reference standard, with assessment metrics including sensitivity, specificity, positive predictive value (PPV), negative predictive value (NPV), and the area under the receiver operating characteristic curve (AUC). The diagnostic value of different LAM/IGRA result patterns for specific diseases was evaluated by analyzing their distribution characteristics among patients with tuberculosis, NTM infection, and non-tuberculous diseases. A two-sided *P* < 0.05 was considered statistically significant for all tests. Data processing and analysis were performed using R software (version 4.2.1) and Zstats v1.0 (www.zstats.net). Graphs comparing LAM concentrations across different groups were generated using GraphPad Prism version 8.

## Results

**Figure 1:**
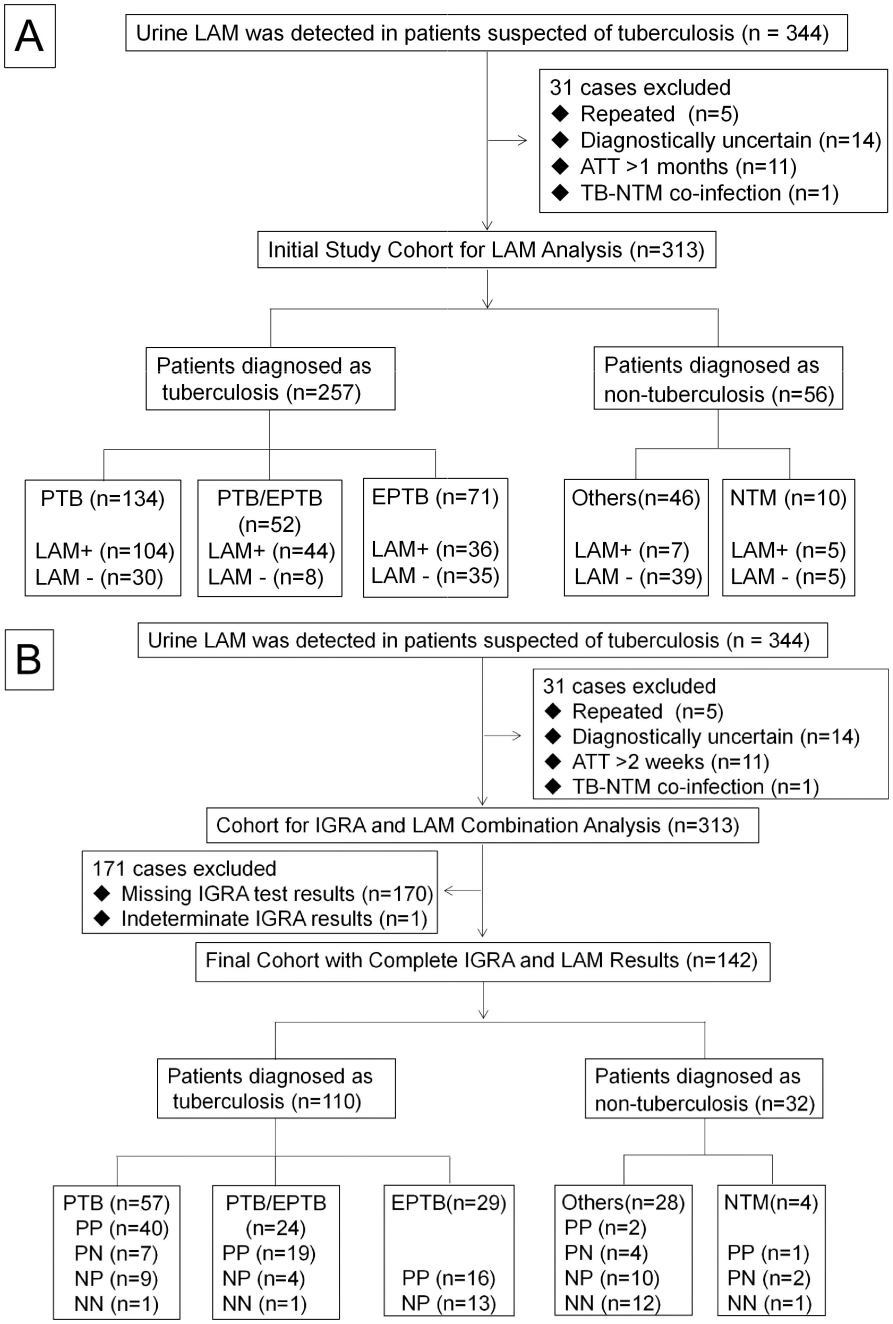
Participant inclusion flowchart for diagnostic evaluation. **(A)** Flowchart of study cohort construction for LAM detection performance evaluation. Notes: Others, community-acquired pneumonia (10 cases), pulmonary infection (9 cases), chronic obstructive pulmonary disease (4 cases), lymphadenitis (2 cases), multi-pathogen pneumonia (2 cases), and lumbar abscess (2 cases). A spectrum of 17 other conditions was represented by one case each. ATT, antituberculosis treatment; PTB, Pulmonary Tuberculosis; EPTB, Extrapulmonary Tuberculosis; PTB/EPTB, Concomitant Pulmonary and Extrapulmonary Tuberculosis; NTM, Nontuberculous Mycobacteria. **(B)** Flowchart of study cohort construction for the combined analysis of IGRA and LAM. Notes: Others, Including community-acquired pneumonia (8 cases), pulmonary infection (5 cases), chronic obstructive pulmonary disease (3 cases), lymphadenitis (2 cases), and a spectrum of other conditions with one case each, including infectious shoulder arthritis, lumbar abscess, pleural effusion, lumbar compression fracture, viral myocarditis, idiopathic pulmonary fibrosis, obstructive pneumonia, chronic bronchitis, chlamydial pneumonia, and pyogenic spondylitis. abbreviations: PP, LAM+/IGRA+; PN, LAM+/IGRA-; NP, LAM-/IGTA+; NN, LAM-/IGRA-.

Between April 2023 and June 2024, a total of 344 patients with suspected TB were initially screened using urine LAM assay. After applying exclusion criteria—duplicate tests (n=5), indeterminate diagnosis (n=14), anti-TB treatment exceeding 2 weeks (n=11), and TB-NTM co-infection (n=1)—a preliminary cohort of 313 patients was established for evaluating the diagnostic performance of LAM assay alone (**Figure 1A**). To further analyze the combined diagnostic value of LAM and IGRA, patients lacking IGRA results (n=170) or with indeterminate IGRA outcomes (n=1) were excluded from the preliminary cohort. The final analysis included 142 patients with complete LAM and IGRA results, comprising 110 active TB cases and 32 non-TB cases (**Figure 1B**). To address potential selection bias, the 313 patients in the preliminary cohort were categorized into two groups based on the availability of complete LAM and IGRA results: an included group (n=142) and an excluded group (n=171). Comparative analysis demonstrated that the two groups were well-balanced across all key baseline clinical characteristics, with no statistically significant differences (*P* > 0.05, **Supplementary Table 1**).

### Comparison of baseline characteristics of the study population

A total of 313 patients were included in this study to evaluate the performance of the LAM test., including 257 TB cases and 56 non-TB cases. The median age of the control group was significantly higher than that of the tuberculosis group (*P* = 0.011), while no significant differences were found in gender (P = 0.695) or lymphocyte count (< 0.8 × 10^9^/L) (P = 0.394). Bronchoalveolar lavage fluid and sputum were the most common specimen types used for etiological diagnosis, with no significant difference in specimen type distribution between groups. The TB group comprised PTB (n = 134), EPTB (n = 71), and PTB/EPTB (n = 52). The control group consisted of patients with Non-TB (n = 46) and those with NTM infections (n = 10) (**Table 1**).

**Table 1:** Baseline characteristics and demographics of participants.

Characteristics	Total	Tuberculosis group	Control group	*P*-value*
	N=313	N=257	N=56	
Age, M (Q_1_;, Q_2_)	60.00 (45.00, 71.00)	59.00 (42.00, 71.00)	67.00 (53.00, 74.00)	0.011
Gender(male), n(%)	194 (61.98)	158 (61.48)	36 (64.29)	0.695
Total lymphocyte count (<0.8×10^9^/L), n(%)	93 (29.71)	79 (30.74)	14 (25.00)	0.394
Diagnostic basisn(%)				<0.001
Etiological Evidence	234 (74.76)	224 (87.16)	10^#^ (17.86)	
Absence of etiological evidence	79 (25.24)	33 (12.84)	46 (82.14)	
Sample type, n(%)				0.105
BALF	106 (33.87)	80 (31.13)	26 (46.43)	
Sputum	89 (28.43)	75 (29.18)	14 (25.00)	
Tissue	50 (15.97)	41 (15.95)	9 (16.07)	
Others	68 (21.73)	61 (19.49)	7 (12.50)	
clinical subgroup n(%)				<0.001
PTB	134 (42.81)	134 (52.14)	0 (0.00)	
EPTB	71 (22.68)	71 (27.63)	0 (0.00)	
PTB/EPTB	52 (16.61)	52 (20.23)	0 (0.00)	
Non-TB	46 (14.70)	0 (0.00)	46 (82.14)	
NTM	10 (3.19)	0 (0.00)	10 (17.86)	

M, Median, Q₁, 1st Quartile, Q₃, 3st Quartile; P-value*, Indicates the comparison between Tuberculosis group and Control group; 10#, 10 cases of NTM etiological diagnosis; Tissue, refers to substantial tissue blocks obtained through biopsy or surgery; Others, Including pus, pleural fluid, puncture fluid, urine, cerebrospinal fluid, pericardial effusion, Necrotic debris, stool, ascites, bone marrow, and arthritis samples; PTB, Pulmonary Tuberculosis; EPTB, Extrapulmonary Tuberculosis; PTB/EPTB, Concomitant Pulmonary and Extrapulmonary Tuberculosis.

### Comparison of urine LAM antigen concentrations across different patient groups

**Figure 2:**
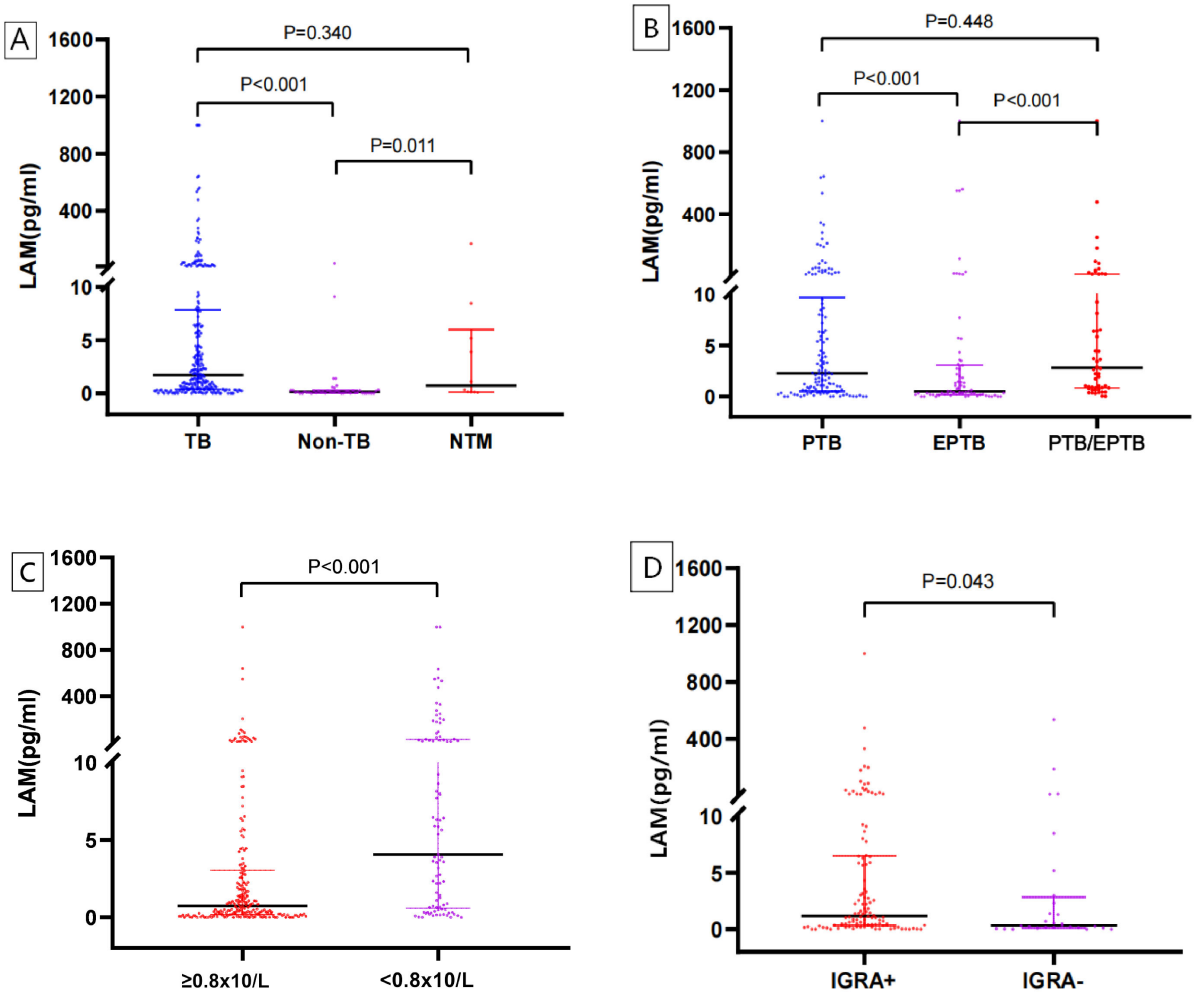
Urine LAM antigen levels across clinical subgroups and patient characteristics. **(A)** Across diagnostic categories, **(B)** Across clinical subgroups, **(C)** According to total lymphocyte count, **(D)** According to IGRA results.

We compared urine LAM antigen concentrations across different patient groups. The results demonstrated that both the TB group and the NTM group exhibited significantly higher median LAM concentrations than the Non-TB group (*P* < 0.001 and P = 0.011, respectively). Although the median LAM concentration in the TB group was higher than that in the NTM group, the difference was not statistically significant (*P* = 0.340; **Figure 2A**). In the clinical subgroup analysis, patients with PTB and those with PTB/EPTB showed significantly higher median LAM concentrations than those with exclusively EPTB (both *P* < 0.001). However, no significant difference was observed between the PTB and PTB/EPTB groups (*P* = 0.448; **Figure 2B**). Further analysis revealed a significant association between immune status and LAM antigen levels. Patients with lymphocyte counts <0.8×10^9^/L had significantly higher LAM concentrations than those with counts ≥0.8×10^9^/L (*P* < 0.001; **Figure 2C**). Concurrently, analysis of the relationship between IGRA results and LAM concentrations indicated that IGRA-positive patients also exhibited significantly higher LAM levels than IGRA-negative individuals (*P* = 0.043; **Figure 2D**).

### Diagnostic performance of the combined LAM and IGRA strategy for TB

This study evaluated the diagnostic performance of urine LAM assay for active TB, demonstrating a sensitivity of 72%, specificity of 79%, and an AUC of 0.75, indicating moderate diagnostic accuracy. Compared with conventional methods, LAM assay exhibited significantly higher sensitivity than smear microscopy (33%) and culture (56%), and was slightly superior to Xpert (69%) and TB-DNA (65%), though lower than IGRA (92%). In contrast, its specificity was lower than smear microscopy (93%), culture (84%), Xpert (100%), and TB-DNA (92%), but higher than IGRA (59%) (**Table 2**). Further analysis of combined LAM and IGRA strategies revealed that a parallel approach (“LAM or IGRA” positive) increased diagnostic sensitivity to 98%, significantly enhancing detection capability for active TB—particularly valuable for screening or rule-out diagnosis in high-risk populations. Conversely, a serial strategy (“LAM and IGRA” positive) elevated specificity to 91% (**Table 3**), supporting confirmatory diagnosis and reducing false positives. Subgroup analysis in etiology-confirmed patients showed increased sensitivity for conventional tests (smear, culture, Xpert, TB-DNA), while sensitivity of LAM, IGRA and their combined strategies remained stable, indicating their retained ability to identify clinically diagnosed cases (**Supplementary Tables 2, 3**). By effectively integrating the complementary strengths of LAM and IGRA, both strategies achieved an optimal balance between sensitivity and specificity, thereby optimizing the clinical diagnostic workflow for TB.

**Table 2:** Diagnostic performance of several tests in tuberculosis patient.

Test	CRS diagnosed		Sensitivity	Specificity	PPV	NPV	AUC
	Positive	Negative	%(95%CI)	%(95%CI)	%(95%CI)	%(95%CI)	%(95%CI)
smear			0.33 (0.26 - 0.39)	0.93 (0.85 - 1.00)	0.96 (0.91 - 1.00)	0.21 (0.15 - 0.27)	0.63 (0.58-0.68)
Positive	71	3					
Negative	147	39					
Culture			0.56 (0.50 - 0.62)	0.84 (0.73 - 0.95)	0.95 (0.92 - 0.99)	0.26 (0.18 - 0.33)	0.70 (0.64-0.76)
Positive	138	7					
Negative	108	37					
Xpert			0.69 (0.63 - 0.75)	1.00 (1.00 - 1.00)	1.00 (1.00 - 1.00)	0.38 (0.30 - 0.47)	0.85 (0.82-0.87)
Positive	177	0					
Negative	79	49					
TB-DNA			0.65 (0.59 - 0.71)	0.92 (0.84 - 1.00)	0.97 (0.95 - 1.00)	0.35 (0.27 - 0.44)	0.78 (0.74-0.83)
Positive	153	4					
Negative	82	45					
IGRA			0.92 (0.87 - 0.97)	0.59 (0.42 - 0.76)	0.89 (0.83 - 0.94)	0.68 (0.51 - 0.85)	0.76 (0.67-0.85)
Positive	101	13					
Negative	9	19					
LAM			0.72 (0.66 - 0.77)	0.79 (0.68 - 0.89)	0.94 (0.91 - 0.97)	0.38 (0.29 - 0.46)	0.75 (0.69-0.81)
Positive	184	12					
Negative	73	44					

CRS, composite reference standard; LAM, lipoarabinomannan; CI, confidence interval; AUC, area under the curve; PPV, Positive predictive value; NPV, Negative predictive value.

**Table 3:** Comparison of IGRA combined with LAM for tuberculosis diagnosis versus several other methods.

Test	CRS diagnosed		Sensitivity	Specificity	PPV	NPV	AUC
	Positive	Negative	%(95%CI)	%(95%CI)	%(95%CI)	%(95%CI)	%(95%CI)
smear			0.30 (0.21 - 0.39)	0.96 (0.87 - 1.00)	0.97 (0.90 - 1.00)	0.24 (0.16 - 0.33)	0.63 (0.57-0.69)
Positive	29	1					
Negative	68	22					
Culture			0.60 (0.51 - 0.69)	0.86 (0.73 - 0.99)	0.94 (0.88 - 1.00)	0.36 (0.25 - 0.48)	0.73 (0.65-0.81)
Positive	63	4					
Negative	42	24					
Xpert			0.62 (0.53 - 0.71)	1.00 (1.00 - 1.00)	1.00 (1.00 - 1.00)	0.41 (0.30 - 0.53)	0.81 (0.77-0.86)
Positive	68	0					
Negative	41	29					
TB-DNA			0.60 (0.50 - 0.69)	0.93 (0.83 - 1.00)	0.97 (0.92 - 1.00)	0.41 (0.29 - 0.53)	0.76 (0.69-0.83)
Positive	56	2					
Negative	38	26					
IGRA			0.92 (0.87 - 0.97)	0.59 (0.42 - 0.76)	0.89 (0.83 - 0.94)	0.68 (0.51 - 0.85)	0.76 (0.67-0.85)
Positive	101	13					
Negative	9	19					
LAM			0.75 (0.66 - 0.83)	0.72 (0.56 - 0.87)	0.90 (0.84 - 0.96)	0.45 (0.31 - 0.59)	0.73 (0.64-0.82)
Positive	82	9					
Negative	28	23					
IGRA and LAM			0.68 (0.59 - 0.77)	0.91 (0.81 - 1.00)	0.96 (0.92 - 1.00)	0.45 (0.33 - 0.58)	0.79 (0.73-0.86)
Positive	75	3					
Negative	35	29					
IGRA or LAM			0.98 (0.96 - 1.00)	0.41 (0.24 - 0.58)	0.85 (0.79 - 0.91)	0.87 (0.69 - 1.00)	0.69 (0.61-0.78)
Positive	108	19					
Negative	2	13					

### Value of combined LAM and IGRA assay in differentiating TB from non-TBs

**Figure 3:**
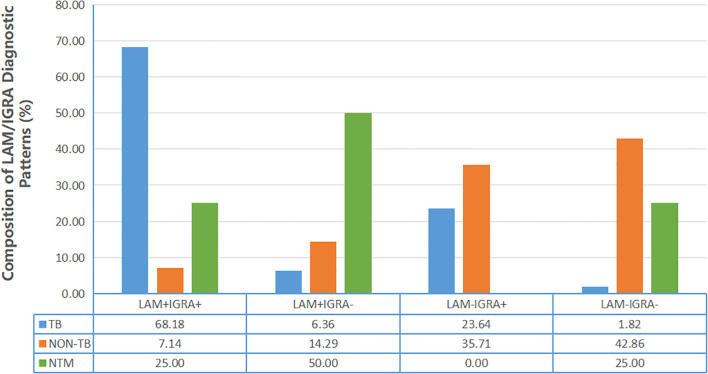
Diagnostic patterns of combined LAM and IGRA testing in active tuberculosis, non-tuberculous diseases, and NTM infection.

This study further analyzed the potential clinical utility of combined LAM and IGRA assay in diagnosing active TB and differentiating it from NTM infection. Among active TB patients, the concurrent positivity pattern (LAM^+^IGRA^+^) was most prevalent (68.18%), demonstrating high diagnostic value for active TB. In contrast, the double-negative pattern (LAM^−^IGRA^−^) was rare (only 1.82%), indicating excellent rule-out value. In the non-TB group, the double-negative pattern accounted for a substantial proportion (42.86%), further supporting the strategy’s applicability for excluding TB infection in clinical practice. Particularly noteworthy, the dissociated LAM^+^IGRA^−^ pattern was observed in 50% of NTM-infected patients, significantly higher than in other subgroups, suggesting this signature may help distinguish NTM infection from TB (**Figure 3**, **Supplementary Table 4**).

## Discussion

This study was designed to address the specific challenges in diagnosing active TB, particularly the diagnostic dilemmas encountered in immunocompromised individuals, patients with paucibacillary or sputum-scarce disease, and those with EPTB. While molecular diagnostic technologies such as Xpert have significantly improved the detection capability of TB, their reliance on sputum samples and substantial laboratory infrastructure requirements present constraints for widespread implementation in resource-limited settings (16). Recognizing this need, the WHO has explicitly advocated for the development of accurate, convenient, and non-sputum-based diagnostic tools (5). Against this backdrop, our study employed a cross-sectional design to systematically evaluate the performance of a chemiluminescence-based urine LAM assay for TB diagnosis, conducting comprehensive comparisons with established laboratory methods. Furthermore, we innovatively proposed a novel diagnostic strategy combining LAM with the IGRA. This integrated approach synergizes pathogen-derived antigen detection with host-specific immune response information, effectively compensating for the limitations of individual tests and substantially enhancing the overall diagnostic performance for TB.

First, analysis of the baseline characteristics revealed no significant differences in lymphocyte counts or specimen type distribution between the TB group and non-TB. This comparability helps minimize potential performance bias in both LAM and IGRA assays that could arise from impaired immune function or variations in specimen sources (13). Furthermore, the TB group in this study included a substantial proportion of patients with EPTB (27.63%) and PTB/EPTB (20.23%). These patient subgroups typically present with low bacterial loads and pose challenges for obtaining definitive pathogen evidence (17), representing a diagnostically challenging population. Consequently, the study cohort provides a representative background well-suited for evaluating the value of novel, non-invasive diagnostic technologies like LAM in real-world clinical settings.

Comparison of urine LAM antigen concentrations across different patient groups revealed significantly elevated levels in both the TB and NTM infection groups compared to the Non-TB group. This finding suggests that LAM assay has potential value in differentiating mycobacterial diseases from Non-TB conditions. However, no statistically significant difference in LAM concentration was observed between the TB and NTM groups, a result consistent with previous studies. This phenomenon may be attributed to the widespread presence of LAM antigen in mycobacterial cell walls and its lack of species specificity (18, 19). In the Clinical subgroup analysis of TB, the median LAM concentrations in both the PTB group and the PTB/EPTB group were significantly higher than those in the EPTB group. This disparity may be attributed to the typically lower bacterial load in extrapulmonary lesions, reduced antigen release into circulation, and potentially different efficiency of urinary antigen excretion. This observation aligns with previously reported findings of generally lower LAM detection rates in EPTB patients (20). Notably, this study further revealed a significant influence of host immune status on urine LAM concentration. Patients with lymphocyte counts <0.8 × 10^9^/L exhibited substantially elevated LAM levels, a finding consistent with the report by Li et al. (13). This suggests that immunosuppression may lead to increased urinary LAM levels, potentially due to impaired antigen clearance and/or enhanced bacterial lysis. This result not only aligns with the biological nature of LAM as a pathogen-derived antigen but also provides a mechanistic rationale for expanding its diagnostic utility in immunocompromised populations (21). Concurrently, we observed significantly higher LAM concentrations in IGRA-positive individuals compared to IGRA-negative subjects. This correlation suggests that higher bacterial antigen load may coexist with robust specific cellular immune responses, thereby supporting the mechanistic complementarity between LAM and IGRA from the perspective of host-pathogen immune interactions.

This study evaluated the diagnostic performance of urine LAM detection for active tuberculosis, showing a sensitivity of 72%, specificity of 79%, and an AUC value of 0.75, indicating moderate diagnostic value. The sensitivity observed in our study was significantly higher than that reported in earlier studies based on the lateral flow assay (LFA). This difference primarily stems from fundamental distinctions in the detection technology principles. Traditional LFA methods rely on visual interpretation of results, which limits analytical sensitivity and is susceptible to subjective factors. In contrast, the high-sensitivity chemiluminescence immunoassay used in our study detects chemiluminescence signals, offering greater analytical sensitivity, a superior signal-to-noise ratio, and precise quantitative capabilities. This enables the effective detection of lower concentrations of LAM antigen in urine, thereby significantly enhancing clinical detection sensitivity. These findings are consistent with those reported by Li et al. (2025) and Huang et al. (2023) using similar chemiluminescence platforms (13, 18, 22), collectively affirming the importance of high-sensitivity detection technologies like CLIA in realizing the diagnostic potential of LAM. However, compared to molecular diagnostic methods such as Xpert, LAM assay still shows a persistent challenge in specificity, particularly in distinguishing between *M. tuberculosis* and NTM infections. This limitation corroborates our earlier observation of comparable LAM concentrations between the NTM and TB groups.

In clinical practice, the combination of multiple diagnostic methods has become a key strategy for improving TB detection rates. Given the specific characteristics and limitations of urine LAM assay in terms of sensitivity and specificity, this study further systematically evaluated the diagnostic value of combining LAM with IGRA. The results demonstrated that a parallel testing strategy (“LAM or IGRA” positive) increased the diagnostic sensitivity to 98%, substantially enhancing screening and rule-out capacity—particularly suitable for initial screening in high-risk populations or complex cases. Conversely, a serial testing strategy (“LAM and IGRA” positive) achieved a specificity of 91%, supporting the confirmation of active TB and reducing false-positive diagnoses. These findings indicate that flexibly selecting the appropriate combined strategy based on different clinical scenarios and diagnostic objectives can significantly optimize the overall diagnostic pathway for TB. Further analysis of different testing combination patterns revealed distinct diagnostic implications. The dual-positive profile (LAM^+^IGRA^+^) demonstrated the highest prevalence among active TB patients (68.18%), indicating its strong diagnostic value for active TB. Conversely, the dual-negative pattern (LAM^−^IGRA^−^) was exceptionally rare in active TB cases (merely 1.82%), underscoring its excellent rule-out utility. Particularly noteworthy, the dissociated LAM^+^IGRA^−^ profile accounted for 50% of NTM infection cases, suggesting this signature may play a crucial role in differentiating between *M. tuberculosis* and NTM infections, thereby revealing promising applications in pathogen discrimination.

This study has several limitations. First, as a single-center, cross-sectional investigation with a limited sample size and lack of multi-center population inclusion, the generalizability of the findings is constrained. Second, the absence of detailed immunological data, such as CD4^+^ T-cell counts, limited our ability to thoroughly explore host immune factors influencing LAM positivity and fully explain the heterogeneity in its diagnostic performance. Third, the limited number of pediatric TB cases precluded evaluation of LAM assay in this population—a key target group for non-sputum-based diagnostics—thereby leaving its applicability in children uncertain. Finally, the small sample size of the NTM infection group restricted accurate assessment of LAM assay performance in NTM cases and the potential influence of species-specific variations on test results.

## Conclusion

The findings of this study demonstrate that combining urine LAM antigen testing with blood-based IGRA significantly enhances the overall diagnostic capacity for active TB. The parallel testing strategy (“LAM or IGRA”) proves suitable for screening high-risk populations or rule-out diagnosis, while the serial approach (“LAM and IGRA”) facilitates confirmatory diagnosis of active TB. This integrated methodology, utilizing both urine and blood samples, capitalizes on non-invasive sampling and operational convenience, thereby offering a novel and efficient diagnostic pathway for challenging TB cases—particularly those involving immunocompromised individuals, sputum smear-negative PTB, and EPTB.

## Data Availability

The original contributions presented in the study are included in the article/**Supplementary Material**. Further inquiries can be directed to the corresponding author.
