# Supplementary material for: A novel non-invasive diagnostic approach based on urine and blood: a cross-sectional study combining lipoarabinomannan antigen and interferon-gamma release assay for active tuberculosis
Source: Front Immunol. 2026 Jan 9;16:1726930. doi: 10.3389/fimmu.2025.1726930 (PMC12827696; doi:10.3389/fimmu.2025.1726930)
Supplement: Supplementary file 1 [file Table1.docx]

Supplementary Table 1. Comparison of Baseline Characteristics Between Included and Excluded Groups in the Combined LAM and IGRA Analysis

| **Variables** | **Total (n = 313)** | **Excluded group**  **(n = 171)** | **Included group**  **(n = 142)** | **P-value*** |
| --- | --- | --- | --- | --- |
|  |  |  |  |  |
| Age, M (Q₁, Q₃) | 60.00 (45.00, 71.00) | 59.00 (42.00, 70.00) | 62.00 (49.00, 73.00) | 0.163 |
| Gender(male), n(%) | 194 (61.98) | 107 (62.57) | 87 (61.27) | 0.813 |
| Clinical subgroup, n(%) |  |  |  | 0.230 |
| PTB | 134 (42.81) | 77 (45.03) | 57 (40.14) |  |
| EPTB | 71 (22.68) | 42 (24.56) | 29 (20.42) |  |
| PTB/EPTB | 52 (16.61) | 28 (16.37) | 24 (16.90) |  |
| Non-TB | 46 (14.70) | 18 (10.53) | 28 (19.72) |  |
| NTM | 10 (3.19) | 6 (3.51) | 4 (2.82) |  |
| Final Diagnosis, n(%) |  |  |  | 0.051 |
| Non-TB | 56 (17.89) | 24 (14.04) | 32 (22.54) |  |
| TB | 257 (82.11) | 147 (85.96) | 110 (77.46) |  |
| Sample type, n(%) |  |  |  | 0.089 |
| BALF | 106 (33.87) | 50 (29.24) | 56 (39.44) |  |
| Sputum | 89 (28.43) | 55 (32.16) | 34 (23.94) |  |
| Tissue | 50 (15.97) | 24 (14.04) | 26 (18.31) |  |
| Others | 68 (21.73) | 42 (24.56) | 26 (18.31) |  |
| LAM, n(%) |  |  |  | 0.625 |
| Negative | 117 (37.38) | 66 (38.60) | 51 (35.92) |  |
| Positive | 196 (62.62) | 105 (61.40) | 91 (64.08) |  |
| Total lymphocyte count (<0.8×10^9^/L), n(%) | 220 (70.29) | 121 (70.76) | 99 (69.72) | 0.841 |

**Note:** M: Median, Q₁: 1st Quartile, Q₃: 3st Quartile; P-value*, Indicates the comparison between Excluded group and Included group; Tissue, refers to substantial tissue blocks obtained through biopsy or surgery; Others, Including pus, pleural fluid, puncture fluid, urine, cerebrospinal fluid, pericardial effusion, Necrotic debris, stool, ascites, bone marrow, and arthritis samples.

Supplementary Table 2. Diagnostic performance of several tests in patients with etiologically confirmed tuberculosis.

| **Test** | **etiologically confirmed tuberculosis** | | **Sensitivity** | **specificity** | **PPV** | **NPV** | **AUC** |
| --- | --- | --- | --- | --- | --- | --- | --- |
|  | **Positive** | **Negative** | **%（95%CI）** | **%（95%CI）** | **%（95%CI）** | **%（95%CI）** | **%（95%CI）** |
| smear |  |  | 0.37 (0.30 - 0.43) | 0.93 (0.85 - 1.00) | 0.96 (0.91 - 1.00) | 0.24 (0.17 - 0.31) | 0.65 (0.60-0.70) |
| Positive | 71 | 3 |  |  |  |  |  |
| Negative | 123 | 39 |  |  |  |  |  |
| Culture |  |  | 0.63 (0.56 - 0.69) | 0.84 (0.73 - 0.95) | 0.95 (0.92 - 0.99) | 0.31 (0.23 - 0.39) | 0.73 (0.67-0.80) |
| Positive | 137 | 7 |  |  |  |  |  |
| Negative | 82 | 37 |  |  |  |  |  |
| Xpert |  |  | 0.79 (0.74 - 0.84) | 1.00 (1.00 - 1.00) | 1.00 (1.00 - 1.00) | 0.51 (0.41 - 0.61) | 0.90 (0.87-0.92) |
| Positive | 177 | 0 |  |  |  |  |  |
| Negative | 47 | 49 |  |  |  |  |  |
| TB-DNA |  |  | 0.73 (0.67 - 0.79) | 0.92 (0.84 - 1.00) | 0.97 (0.95 - 1.00) | 0.44 (0.34 - 0.54) | 0.82 (0.77-0.87) |
| Positive | 153 | 4 |  |  |  |  |  |
| Negative | 57 | 45 |  |  |  |  |  |
| IGRA |  |  | 0.93 (0.88 - 0.99) | 0.59 (0.42 - 0.76) | 0.87 (0.80 - 0.93) | 0.76 (0.59 - 0.93) | 0.76 (0.67-0.85) |
| Positive | 85 | 13 |  |  |  |  |  |
| Negative | 6 | 19 |  |  |  |  |  |
| LAM |  |  | 0.73 (0.67 - 0.79) | 0.79 (0.68 - 0.89) | 0.93 (0.89 - 0.97) | 0.42 (0.33 - 0.52) | 0.76 (0.70-0.82) |
| Positive | 164 | 12 |  |  |  |  |  |
| Negative | 60 | 44 |  |  |  |  |  |

Supplementary Table 3. Comparison of IGRA Combined with LAM for Tuberculosis Diagnosis Versus Several Other Methods in patients with etiologically confirmed tuberculosis.

| **Test** | **etiologically confirmed tuberculosis** | | **Sensitivity** | **specificity** | **PPV** | **NPV** | **AUC** |
| --- | --- | --- | --- | --- | --- | --- | --- |
|  | **Positive** | **Negative** | **%（95%CI）** | **%（95%CI）** | **%（95%CI）** | **%（95%CI）** | **%（95%CI）** |
| smear |  |  | 0.35 (0.25 - 0.45) | 0.96 (0.87 - 1.00) | 0.97 (0.90 - 1.00) | 0.29 (0.19 - 0.39) | 0.65 (0.59-0.72) |
| Positive | 29 | 1 |  |  |  |  |  |
| Negative | 54 | 22 |  |  |  |  |  |
| Culture |  |  | 0.70 (0.61 - 0.79) | 0.86 (0.73 - 0.99) | 0.94 (0.88 - 1.00) | 0.47 (0.33 - 0.61) | 0.78 (0.70-0.86) |
| Positive | 63 | 4 |  |  |  |  |  |
| Negative | 27 | 24 |  |  |  |  |  |
| Xpert |  |  | 0.75 (0.66 - 0.84) | 1.00 (1.00 - 1.00) | 1.00 (1.00 - 1.00) | 0.56 (0.42 - 0.69) | 0.87 (0.83-0.92) |
| Positive | 68 | 0 |  |  |  |  |  |
| Negative | 23 | 29 |  |  |  |  |  |
| TB-DNA |  |  | 0.68 (0.58 - 0.78) | 0.93 (0.83 - 1.00) | 0.97 (0.92 - 1.00) | 0.50 (0.36 - 0.64) | 0.81 (0.74-0.88) |
| Positive | 56 | 2 |  |  |  |  |  |
| Negative | 26 | 26 |  |  |  |  |  |
| IGRA |  |  | 0.93 (0.88 - 0.99) | 0.59 (0.42 - 0.76) | 0.87 (0.80 - 0.93) | 0.76 (0.59 - 0.93) | 0.76 (0.67-0.85) |
| Positive | 85 | 13 |  |  |  |  |  |
| Negative | 6 | 19 |  |  |  |  |  |
| LAM |  |  | 0.76 (0.67 - 0.85) | 0.72 (0.56 - 0.87) | 0.88 (0.81 - 0.96) | 0.51 (0.37 - 0.66) | 0.74 (0.65-0.83) |
| Positive | 69 | 9 |  |  |  |  |  |
| Negative | 22 | 23 |  |  |  |  |  |
| IGRA and LAM |  |  | 0.69 (0.60 - 0.79) | 0.91 (0.81 - 1.00) | 0.95 (0.90 - 1.00) | 0.51 (0.38 - 0.64) | 0.80 (0.73-0.87) |
| Positive | 63 | 3 |  |  |  |  |  |
| Negative | 28 | 29 |  |  |  |  |  |
| IGRA or LAM |  |  | 1.00 (1.00 - 1.00) | 0.41 (0.24 - 0.58) | 0.83 (0.76 - 0.90) | 1.00 (1.00 - 1.00) | 0.70 (0.62-0.79) |
| Positive | 91 | 19 |  |  |  |  |  |
| Negative | 0 | 13 |  |  |  |  |  |

Supplementary Table 4. Positive Rates of Combined LAM and IGRA Test in Different Patient Groups

| **Mode of Combination** | **TB** | **NON-TB** | **NTM** |
| --- | --- | --- | --- |
| LAM+IGRA+ | 68.18%(75/110) | 7.14%(2/28 ) | 25.00%(1/4 ) |
| LAM+IGRA- | 6.36%(7/110 ) | 14.29%(4/28 ) | 50.00% (2/4) |
| LAM-IGRA+ | 23.64%(26/110 ) | 35.71%(10/28 ) | 0.00%(0/4) |
| LAM-IGRA- | 1.82%(2 /110) | 42.86%(12/28 ) | 25.00%(1/4) |
